# Night Suit for Physicians

**Published:** 1881-04

**Authors:** 


Night Suit for Physicians.—Some one, it is stated, has invented a night suit, which a physician can don in a minute and a half, on receiving an urgent call at night. No description of the suit has met our eye, but we conclude it must be exceedingly primitive, in its simplicity, to require so little time between rising from bed and being ready for the street. The proverbial Georgia Major's uniform of Colonial times, consisting of a shirt collar and pair of spurs could hardly be donned more expeditiously.

